# Evolution of forelimb musculoskeletal function across the fish-to-tetrapod transition

**DOI:** 10.1126/sciadv.abd7457

**Published:** 2021-01-22

**Authors:** J. L. Molnar, J. R. Hutchinson, R. Diogo, J. A. Clack, S. E. Pierce

**Affiliations:** 1 Anatomy Department, New York Institute of Technology College of Osteopathic Medicine, Northern Boulevard, Old Westbury, NY 11568, USA.; 2 Structure & Motion Laboratory, Department of Comparative Biomedical Sciences, The Royal Veterinary College, Hawkshead Lane, North Mymms, Hertfordshire AL9 7TA, UK.; 3 Anatomy Department, Howard University College of Medicine, 520 W St. NW, Numa Adams Building, Washington, DC 20059, USA.; 4 University Museum of Zoology, Department of Zoology, University of Cambridge, Downing Street, Cambridge CB2 3EJ, UK.; 5 Museum of Comparative Zoology and Department of Organismic and Evolutionary Biology, Harvard University, Cambridge, MA 02138, USA.

## Abstract

One of the most intriguing questions in vertebrate evolution is how tetrapods gained the ability to walk on land. Although many hypotheses have been proposed, few have been rigorously tested using the fossil record. Here, we build three-dimensional musculoskeletal models of the pectoral appendage in *Eusthenopteron*, *Acanthostega*, and *Pederpes* and quantitatively examine changes in forelimb function across the fin-to-limb transition. Through comparison with extant fishes and tetrapods, we show that early tetrapods share a suite of characters including restricted mobility in humerus long-axis rotation, increased muscular leverage for humeral retraction, but not depression/adduction, and increased mobility in elbow flexion-extension. We infer that the earliest steps in tetrapod forelimb evolution were related to limb-substrate interactions, whereas specializations for weight support appeared later. Together, these results suggest that competing selective pressures for aquatic and terrestrial environments produced a unique, ancestral “early tetrapod” forelimb locomotor mode unlike that of any extant animal.

## Teaser

Musculoskeletal models of the first vertebrate limbs reveal adaptations for propulsion, but not weight support.

## INTRODUCTION

The evolution of terrestrially capable tetrapod limbs from aquatically adapted fins has inspired decades of scientific investigation. Paleontological and developmental studies have explained how sarcopterygian fin bones gave rise to tetrapod limb bones [e.g., (*1*–*7*) and references therein], but controversies remain about where and how the earliest tetrapods used their limbs. For example, animals as disparate as mudskippers, salamanders, and seals have been proposed as models for terrestrial locomotion in early tetrapods [e.g., (*8*–*11*); for simplicity, we use the apomorphy-based definition of “Tetrapoda,” a clade defined by the presence of limbs; (*12*)]. Many studies have argued that the first known tetrapods were fully aquatic (*2*, *6*), but others contended that some were capable of moving on land (*9*, *13*). Although previous work on the locomotion of early tetrapods focused mainly on the skeleton (*6*, *8*, *9*, *14*–*19*) and fossil footprints (e.g., *13*, *20*), recent attempts to model soft tissue in early tetrapods have provided valuable information about locomotion, mode of life, and ecology (*21*, *22*).

Multiple locomotor hypotheses have been proposed on the basis of analysis of the early tetrapod fossil record, ranging from underwater walking to fully terrestrial quadrupedal gaits and various modes in between (*10*). For example, because the earliest (Devonian) tetrapods retained features such as lateral line canals and large tail fins, it has been suggested they lacked terrestrial ability and instead used their limbs for underwater walking and station holding (*14*). However, at least one Devonian tetrapod, *Ichthyostega*, may have been able to move on land using a forelimb-driven “crutching” gait (*9*, *17*). In contrast, recent analyses of Devonian-aged tetrapod trackways have contended that the earliest limbed vertebrates used tetrapod-like quadrupedal walking either in shallow water or on land (*20*). Terrestrial walking gaits have also been posited for various early Carboniferous tetrapods based on, e.g., forward-pointing feet (*15*) and a partially healed fracture seemingly incurred during a fall on land (*23*). It has also been proposed that early tetrapods evolved through a phase of underwater walking (*11*) or forelimb-driven belly dragging (*24*), but this idea has yet to be tested using fossil material.

**Fig. 1:**
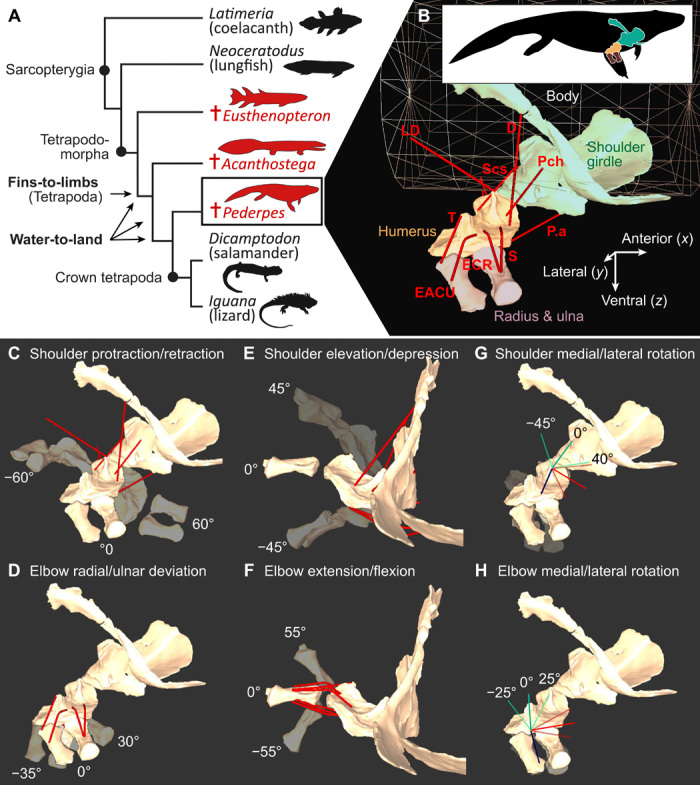
Relationships of study taxa and example musculoskeletal model showing definitions of forelimb movements. (**A**) Cladogram showing relationships between extinct (†) and extant taxa and the node defining the fin-to-limb and hypothesized water-to-land transitions. Three fossil taxa with well-preserved appendages were sampled: the finned tetrapodomorph *Eusthenopteron foordi*, the Devonian tetrapod *Acanthostega gunnari*, and the Carboniferous tetrapod *Pederpes finneyae*. The extant taxa included in the study were chosen as representative examples of the two closest sister groups of tetrapods (Actinistia and Dipnoi) and the two major clades of extant tetrapods (Amniota and Lissamphibia). (**B**) Musculoskeletal model of the pectoral appendage of *Pederpes* in dorsolateral view, showing bony elements (shoulder girdle, humerus, radius, and ulna), cylinder representing the body profile, and reconstructed muscle paths (red lines). (**C** to **H**) *Pederpes* model in dorsolateral (C, D, G, and H) and anterior (E and F) views showing definitions of forelimb movements at the glenohumeral (shoulder) and humeroradioulnar (elbow) joints. Colored lines in (G) indicate axes for long-axis rotation (blue), elevation/depression (red), and protraction/retraction (green). Colored lines in (H) indicate axes for long-axis rotation (blue), flexion/extension (red), and radial/ulnar deviation (green). mm. D, deltoid; EACU, extensor antebrachii et carpi ulnaris; ECR, extensor carpi radialis; LD, latissimus dorsi; P.a, pectoralis anterior; Pch, procoracohumeralis; Scs, subcoracoscapularis; S, supinator; T, triceps. See figs. S1 and S2 and movies S1 to S3 for musculoskeletal models of all taxa sampled. See Materials and Methods for model construction.

Taking advantage of our recent work using extant phylogenetic bracketing to reconstruct appendicular anatomy across the fin-to-limb transition (*21*, *22*), we approach the question of early tetrapod limb function using three-dimensional musculoskeletal modeling, which has been used previously to investigate locomotion in extinct hominids and dinosaurs [e.g., (*25*, *26*)] but never before in early tetrapods. To reconstruct musculoskeletal function and its impact on locomotor evolution, we compare osteological range of motion (ROM) and muscle leverage in the pectoral appendages of an extinct finned tetrapodomorph [*Eusthenopteron foordi*; (*27*)] and two early tetrapods [*Acanthostega gunnari* (*2*, *28*) and *Pederpes finneyae* (*15*)] with two extant finned sarcopterygians (lungfish and coelacanth) and two extant tetrapods (salamander and lizard; Fig. 1 and table S1). As a measure of maximum joint mobility, osteological ROM limits the poses an animal could have assumed in life (*29*). Muscle leverage (quantified by moment arms) influences the maximum rotational force and velocity of movements of a joint, or the ability to stabilize a joint against motion [e.g., (*30*)]. Together, these metrics can reveal trade-offs in the locomotor system such as stability versus mobility and limb forces versus arcs of movement, allowing us to test functional hypotheses in extinct animals. Our results show a combination of maximum osteological ROM and muscle leverage in the forelimb of early tetrapods that is distinct from that of both extant finned sarcopterygians and modern tetrapods, leading us to infer a unique form of locomotor specialization for living at the interface between water and land.

## RESULTS

### Extant fishes versus extant tetrapods

**Fig. 2:**
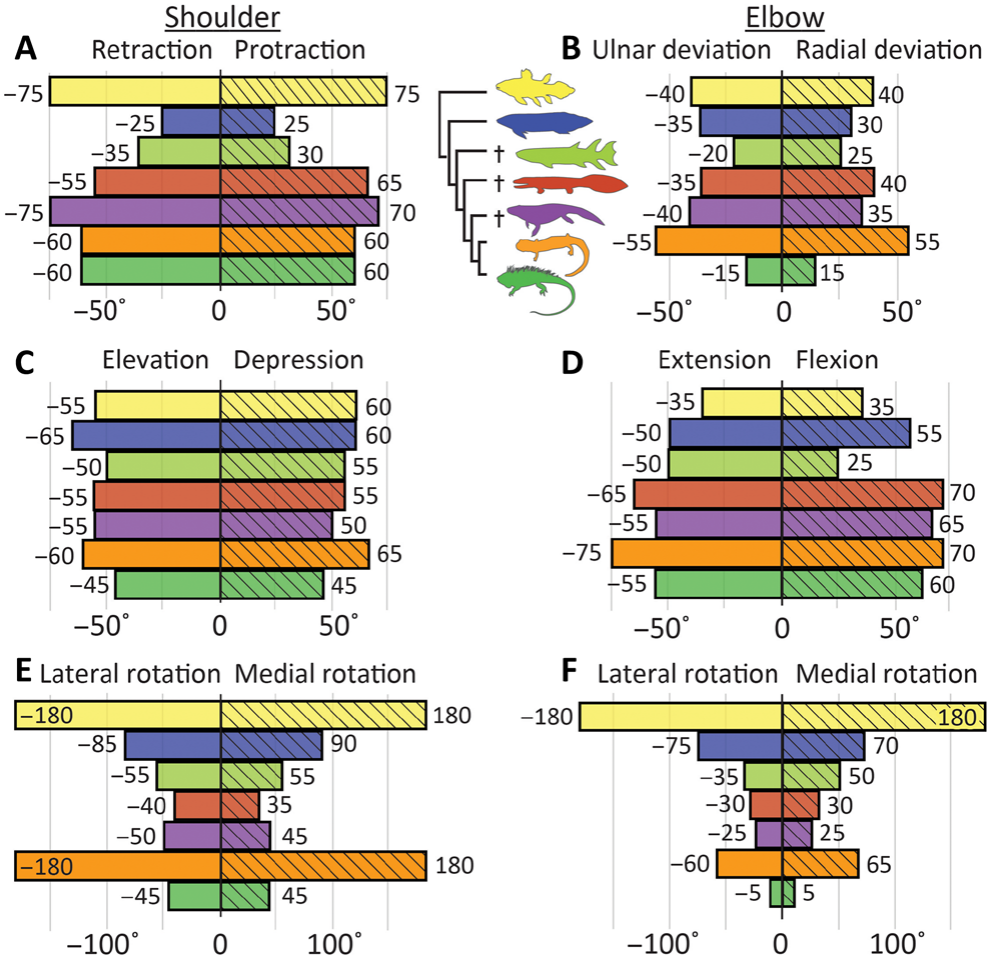
Maximum osteological ROM in the shoulder (glenohumeral) and elbow (humeroradioulnar) joints. (**A**) Shoulder protraction/retraction, (**B**) elbow radial/ulnar deviation, (**C**) shoulder elevation/depression, (**D**) elbow extension/flexion, (**E**) shoulder lateral/medial rotation, and (**F**) elbow lateral/medial rotation. Colors of bars correspond to taxa in the legend; from top to bottom: *Latimeria*, *Neoceratodus*, *Eusthenopteron*, *Acanthostega*, *Pederpes*, *Dicamptodon*, and *Iguana*. Zero represents neutral position (midpoint of osteological ROM without accounting for translation). After accounting for cartilage and a small amount of translation (see Materials and Methods), overall osteological ROM in most taxa in most directions was fairly large, indicating that shoulder and elbow movements were not tightly constrained by either bony stops or disarticulation. Except for *Dicamptodon*, the tetrapods had smaller ROM in long-axis rotation than the fish in both the shoulder and elbow joints. The tetrapod elbow had its largest ROM in flexion/extension. See fig. S5 for results from sensitivity analysis.

**Fig. 3:**
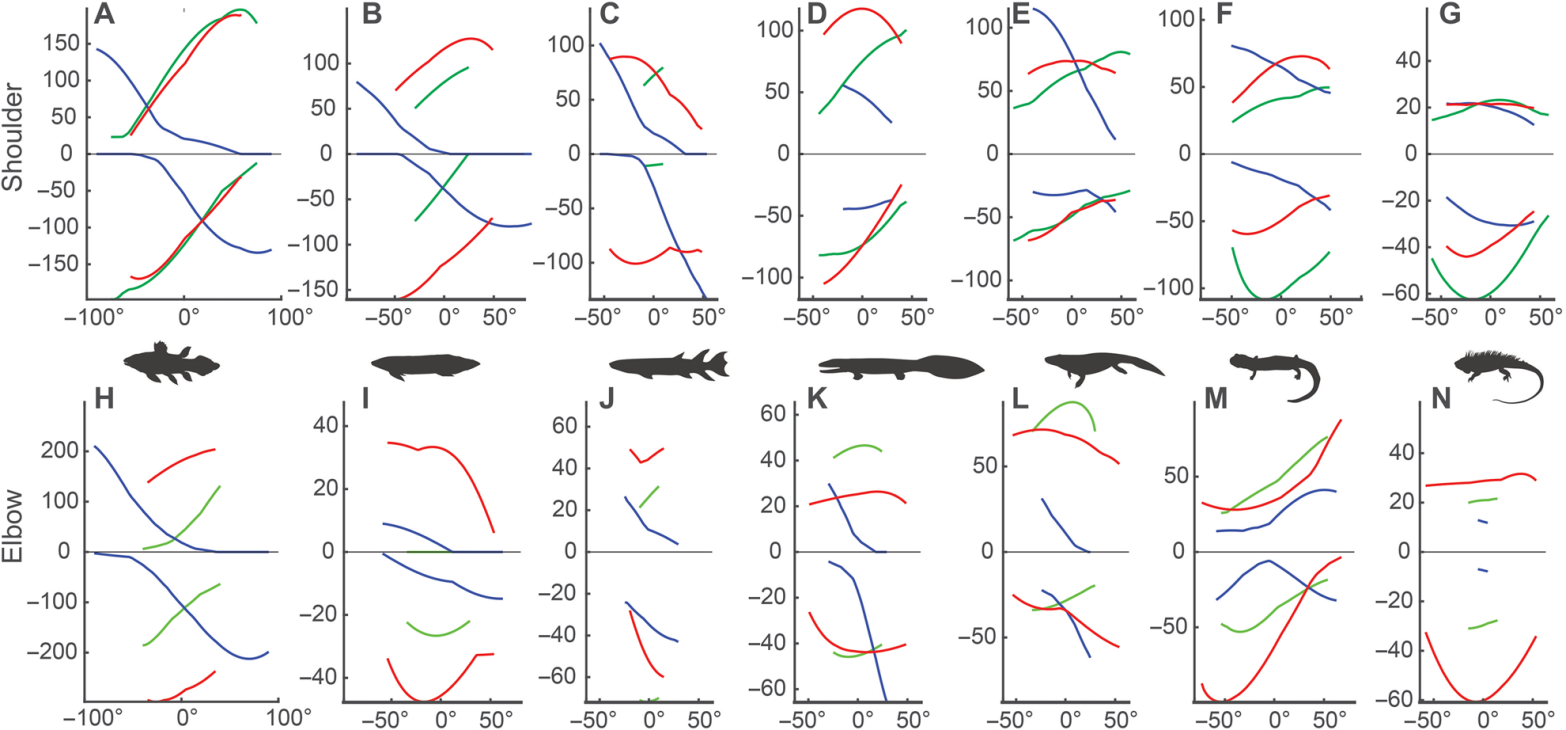
Evolution of shoulder and elbow joint muscle moment arms (leverage) over the tetrapod fin-to-limb transition. (**A** to **G**) Shoulder joint summed moment arms (normalized to humerus length) in protraction (+) and retraction (−) (green), elevation (+) and depression (−) (red), and lateral rotation (+) and medial rotation (−) (blue). (**H** to **N**) Elbow joint summed moment arms (normalized to humerus length) in radial deviation (+) and ulnar deviation (−) (green), extension (+) and flexion (−) (red), and lateral rotation (+) and medial rotation (−) (blue). *X* axis represents joint position, with 0 representing neutral position, positive and negative values corresponding to the same direction, and axis as the curves; e.g., a point on the red curve at *x* = 50 is the result at 50° of elevation. A maximum of 180° in any one axis was imposed to simplify modeling (see Materials and Methods). Taxa are shown by the silhouette in each column: *Latimeria* (A and H), *Neoceratodus* (B and I), *Eusthenopteron* (C and J), *Acanthostega* (D and K), *Pederpes* (E and L), *Dicamptodon* (F and M), and *Iguana* (G and K). Leverage for long-axis rotation and shoulder retraction show a relative increase between *Eusthenopteron* and *Pederpes*, but shoulder depression does not. Crown tetrapods tend to have greater leverage for “stance phase” actions (depression, medial rotation, and retraction). See fig. S4 for moment arms of individual muscles.

On the basis of osteological ROM and muscle leverage, we were able to identify functional differences between fins and limbs (Figs. 2 and 3). At the glenohumeral (shoulder) joint, extant fishes (the coelacanth *Latimeria chalumnae* and the lungfish *Neoceratodus forsteri*) have smaller muscle leverage in humeral lateral (external) rotation than in other directions [Fig. 3, A and B; see Fig. 1 (C to H), movies S1 to S3, and Materials and Methods for definitions of joints and movements]. In the extant tetrapods (the salamander *Dicamptodon ensatus* and the lizard *Iguana iguana*), muscle leverages for humeral lateral rotation, protraction, and elevation (abduction) are similar (Fig. 3, F and G). All else being equal, muscles with greater leverage produce stronger but smaller joint rotations (*30*), so we interpret this result to indicate that the fishes use larger, quicker rotary movements of the fins for functions such as steering. In contrast, humeral lateral rotation in tetrapods is part of a combination of “swing phase” actions, including elevation and protraction, which reposition the limb for the supportive and propulsive (stance) phase of a stride (*31*). The extant tetrapods also have relatively greater muscle leverage for humeral retraction (Fig. 3, F and G), a “stance phase” action (*31*). This result implies that in extant tetrapods, the humeral retractors [which include mm. coracobrachialis, pectoralis posterior, and the posterior part of m. latissimus dorsi; (*32*, *33*)] are comparatively specialized for force production and stabilization.

The tetrapod humeroradioulnar (elbow) joint shows specializations for stabilization through reducing rotation. There is a trend toward increasing ROM in flexion-extension combined with a decrease in long-axis rotation (Fig. 2), which may be related to development and expansion of the ulnar olecranon process in tetrapods (*16*). In *Dicamptodon* and other salamanders, the olecranon process is mostly cartilaginous, so to be consistent with the extinct taxa, we did not model the cartilaginous portion. Had we done so, the trend toward restriction of elbow long-axis rotation in tetrapods would be even more pronounced (see Materials and Methods for determination of limits to osteological ROM). Patterns of leverage in muscles that cross the elbow joint are also different in extant fishes and tetrapods. Fishes have fewer muscles that cross the elbow compared with tetrapods, resulting in zero leverage for some motions in some positions (Fig. 3, H and I). In addition, because the tetrapod humerus is proximodistally twisted, elbow flexion-extension is produced by different muscles in extant lobed-finned fishes and tetrapods. Namely, the dorsal fin m. adductor superficialis extends the “elbow” in *Latimeria* and *Neoceratodus*, whereas mm. brachioradialis and extensor carpi radialis [also a derivative of m. adductor superficialis; (*21*)] flex the elbow in *Dicamptodon* and *Iguana* (fig. S4). As a result, the only muscle modeled in the extant tetrapods with consistent leverage for elbow extension is m. triceps [also a derivative of m. adductor superficialis; (*21*)]. Like the stance phase humeral retractor muscles, m. triceps has a large extension moment arm and appears to be specialized for force production and stabilization, including counteracting the elbow flexion moment produced by the ground reaction force during stance (fig. S5) (*34*).

On the basis of these results, we propose the following characteristics as indicators of crown tetrapod–like forelimb function: at the shoulder joint, greater relative muscle leverage for humeral retraction and similar leverage for humeral protraction, elevation, and lateral rotation (swing phase actions); and at the elbow joint, ROM primarily devoted to flexion-extension and with restricted long-axis rotation, along with m. triceps as the only consistent elbow extensor.

### Differences among extant fishes and among extant tetrapods

Our results also hint at more subtle behavioral signals. The two extant lobed-finned fishes differ greatly in ecology and locomotion: *Latimeria* is a deep-sea pelagic fish that mainly uses its paired fins for stabilization and turning (*35*), whereas *Neoceratodus* is a bottom-dweller that mainly uses its pectoral fins for propping and propulsion during slow swimming (*36*). At the shoulder joint in *Latimeria*, muscle leverage in humerus elevation-depression is similar to protraction-retraction (Fig. 3A), possibly related to the pelagic lifestyle of this fish. Further, *Latimeria* displays no osteological restriction on shoulder or elbow joint long-axis rotation (Fig. 2, E and F), which may be related to the unusually large rotary movements of the pectoral fins (up to 180°) (*35*). In *Neoceratodus*, elevation-depression muscle leverage exceeds protraction-retraction leverage at both the shoulder and elbow joints (Fig. 3, B and I), and humeral protraction-retraction ROM is highly restricted (Fig. 2A), possibly related to its benthic lifestyle (propping its body on the substrate; low activity level). Thus, greater elevation-depression muscle leverage and restricted protraction-retraction ROM at the shoulder may be a signal of fin-substrate interactions.

Both of the extant tetrapods studied (salamanders and lizards) typically use a lateral sequence walk or trot on land (*37*), but some lizards are capable of faster locomotion [with limb duty factors of ~25%; (*38*)]. Relative to other directions, muscle leverage for humeral depression (adduction) is somewhat greater in the lizard than the salamander (averaging approximately 75% of retraction leverage in the former and about 50% in the latter; Fig. 3, F and G), possibly accommodating higher peak forces during fast locomotion. In contrast, muscle leverage for humeral elevation (abduction) is smaller in the lizard (Fig. 3G), possibly allowing it to move its limb faster during the swing phase. Therefore, a larger ratio of humeral depression leverage to other directions of movement (combined with large pro/retraction ROM) may be an indicator of faster terrestrial limb-based locomotion.

### Finned tetrapodomorph *E. foordi*

**Fig. 4:**
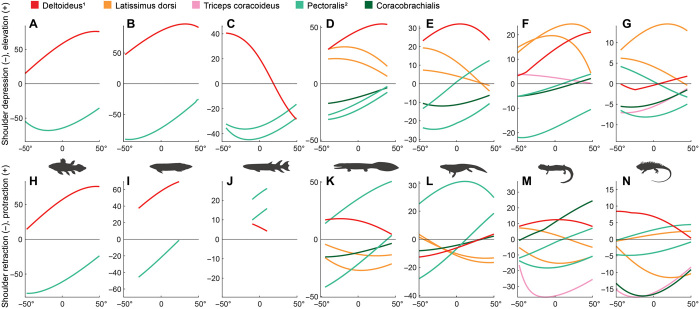
Change in (normalized) moment arms (leverage) of selected individual muscles that cross the shoulder joint. (**A** to **G**) Leverage in elevation (+) and depression (−). (**H** to **N**) Leverage in protraction (+) and retraction (−). Axes and taxa as in Fig. 3. 1 “Deltoideus” in fish (A to C and H to J) corresponds to the proximal part of m. adductor superficialis. 2 “Pectoralis” in fish (A to C and H to J) corresponds to the proximal part of m. abductor superficialis, and in all fossil taxa and extant tetrapods (C to G and J to N), it was modeled with separate anterior and posterior parts (see Materials and Methods). Changes in overall leverage were produced by the differentiation of ancestral muscle masses into multiple individual muscles in more crownward taxa (e.g., mm. latissimus dorsi and deltoideus from adductor superficialis, and mm. coracobrachialis and pectoralis from abductor superficialis in early tetrapods) and by changes in leverage of existing muscles (m. *pectoralis* changed from a retractor to a protractor in tetrapodomorphs, and m. coracobrachialis gained leverage in retraction in *Iguana*). See fig. S4 for moment arms of all individual muscles.

**Fig. 5:**
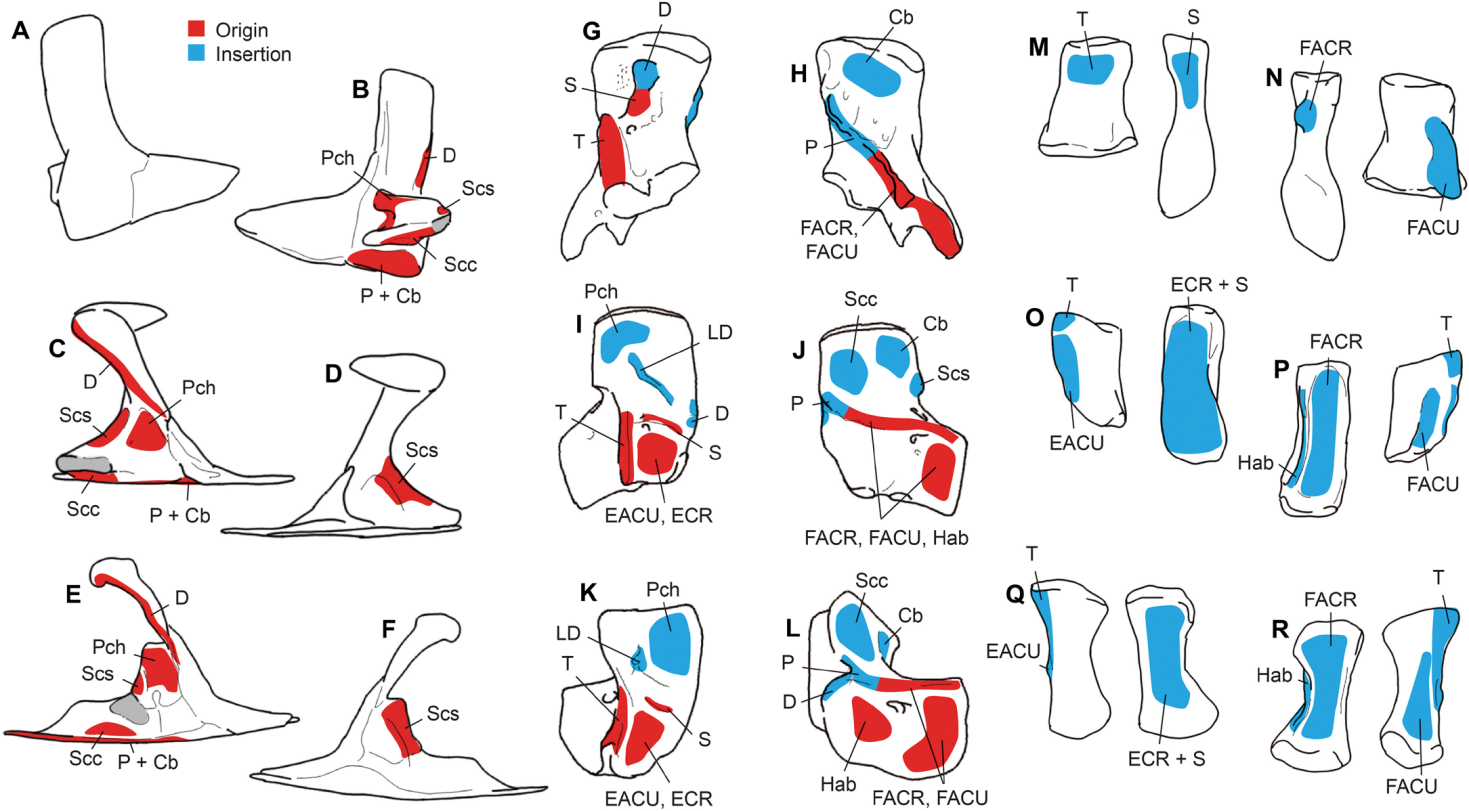
Muscle maps of extinct taxa showing reconstructed areas of origin and insertion. Origins (red) and insertions (blue) are shown for muscles that span the shoulder and/or elbow joints, which were used to build musculoskeletal models. Pectoral girdle in lateral and medial views from *Eusthenopteron* (**A** and **B**, respectively), *Acanthostega* (**C** and **D**), and *Pederpes* (**E** and **F**) (glenoid shown in gray); humerus in dorsal and ventral views from *Eusthenopteron* (**G** and **H**, respectively), *Acanthostega* (**I** and **J**), and *Pederpes* (**K** and **L**); radius and ulna in dorsal and ventral views from *Eusthenopteron* (**M** and **N**, respectively), *Acanthostega* (**O** and **P**), and *Pederpes* (**Q** and **R**). The early tetrapods have a larger number of distinct muscle attachment areas, reflecting the segmentation of large flexor and extensor muscle masses into individual muscles. Cb, mm. coracobrachialis; D, deltoideus; EACU, extensor antebrachii et carpi ulnaris; ECR, extensor carpi radialis; FACR, flexor antebrachii et carpi radialis; FACU, flexor antebrachii et carpi ulnaris; Hab, humeroantebrachialis; LD, latissimus dorsi; P, pectoralis; Pch, procoracohumeralis; Scs, subcoracoscapularis; S, supinator; Scc, supracoracoideus; T, triceps brachii. Reconstructions based on previous studies (*21*) (see Materials and Methods). See fig. S6 for muscle maps of extant taxa.

Despite profound differences from extant finned sarcopterygians in pectoral girdle morphology and glenohumeral shape (*27*) and the number of muscles that cross the shoulder joint (*21*), the pectoral fin of *Eusthenopteron* is, according to our metrics, functionally similar to that of extant lungfish. As in the lungfish, humeral protraction-retraction ROM is highly restricted in *Eusthenopteron* (Fig. 2A), possibly related to a more benthic lifestyle. The degree of long-axis ROM at the shoulder joint of *Eusthenopteron* is intermediate between that of the extant lungfish *Neoceratodus* and the early tetrapod *Acanthostega*, and, similar to *Neoceratodus*, it has relatively little muscle leverage for humeral lateral rotation and retraction (Fig. 3C), implying a capacity for large, quick movements but smaller torques. The small-muscle leverage for humeral retraction in *Eusthenopteron* is mainly caused by the preaxial (i.e., radial) position of the m. pectoralis insertion (Fig. 5H), which changes this muscle’s leverage from retraction (as in the extant fishes) to protraction (Fig. 4J). In addition to protraction, m. pectoralis also has substantial leverage for humeral depression because of the large size of the ventral process of the humerus where it inserts (Fig. 5H). M. pectoralis makes a major contribution toward the relatively large leverage for humeral depression in *Eusthenopteron* (Fig. 3C) that, as mentioned concerning *Neoceratodus*, may be related to propping the body on the substrate. Therefore, our results fall in line with prior ideas that *Eusthenopteron* used its fins in a similar manner to extant lungfish: in slow swimming, turning, and braking, and possibly also for propping its body on the substrate (*27*). A benthic lifestyle has also been attributed to other finned Devonian tetrapodomorphs such as the rhizodontids *Gooloogongia* (*39*) and *Sauripterus* (*40*) on the basis of body form and fin structure. Hence, our results from the pectoral fin support an ancestrally more benthic, lungfish-like lifestyle for tetrapodomorphs such as *Eusthenopteron*.

### Devonian tetrapod *A. gunnari*

Our results depict forelimb musculoskeletal function in *Acanthostega* as a mixture of fish-like and crown tetrapod–like patterns, as well as some patterns apparently unique to early tetrapods. Shoulder joint ROM in protraction-retraction and elevation-depression is comparable to extant tetrapods, but ROM in long-axis rotation is smaller than in any other taxon we studied (Fig. 2). This result is consistent with the idea that the origin of tetrapod limbs coincided with an initial stage of restricted shoulder and hip joint long-axis rotation [e.g., *Ichthyostega* (*9*, *41*)], potentially limiting limb mobility and preventing early tetrapods from using symmetrical quadrupedal gaits such as a lateral-sequence walk or trot. However, our models indicate that shoulder joint long-axis ROM in *Acanthostega* is similar enough to *Iguana* (70° versus 90°, respectively) that the use of tetrapod-like forelimb motions cannot be excluded on this basis (see also fig. S5 for ROM sensitivity analysis). Furthermore, shoulder joint function of *Acanthostega* resembles that of fishes in having much smaller muscle leverage for humeral lateral rotation relative to other directions of movement (Fig. 3D). Therefore, the shoulder joint of *Acanthostega* has a distinctive combination of relatively modest ROM in long-axis rotation and little leverage for this movement. We infer that the restrictive bony structure of the shoulder joint in the earliest tetrapods (Fig. 5, C and E) helped to stabilize the humerus against twisting forces in the absence of high-leverage stabilizing muscles.

In *Acanthostega*, there is a marked increase in muscle leverage for humeral retraction compared with *Eusthenopteron*, both in absolute terms and relative to other directions (Fig. 3, C and D). We interpret this result as indicating an increased ability to generate force against a substrate, either underwater or on land. Interaction between the limb and a substrate during locomotion likely requires more forceful limb extension/retraction (as represented by leverage) than swimming because water is more compliant than a solid surface (*42*). However, humeral retraction leverage in *Acanthostega* is still relatively smaller than in extant tetrapods (Fig. 3, F and G). The increase in humeral retractor muscle leverage in *Acanthostega* is largely produced by m. latissimus dorsi, a large, superficial muscle that is active in lizards and salamanders during both swing and stance (*32*–*34*). This muscle probably first differentiated from its ancestral muscle mass in Devonian tetrapods (*21*), and it contributes substantially to total leverage in both long-axis rotation and retraction of the humerus in our models (Fig. 4 and fig. S3).

The elbow joint of *Acanthostega* also displays a combination of fish-like and tetrapod-like characteristics. As in the extant tetrapods, particularly *Iguana*, the elbow joint in *Acanthostega* has greater ROM in flexion-extension than in any other direction of movement (Fig. 2). This result accords with the notion that *Acanthostega* was among the earliest tetrapods to acquire a habitually flexed elbow and use higher-amplitude flexion-extension movements (*16*). While long-axis rotation at the shoulder joint is restricted by its osteological structure, *Acanthostega* has little bony restriction on elbow movements (Fig. 5, O and P) and would have relied upon cartilage, tendons, and ligaments to reinforce the elbow joint to a greater extent than tetrapods with a larger ossified olecranon process [e.g., *Ichthyostega*, *Tulerpeton*, and more crownward tetrapods such as temnospondyls and reptiliomorphs; (*9*, *16*, *43*–*45*)]. Overall patterns of elbow joint muscle leverage in *Acanthostega* are not very different from those of the extant tetrapods (Fig. 3). Because the humerus of *Acanthostega* lacks the torsion of modern tetrapod humeri (Fig 5, I and J), ventral forearm muscles (e.g., flexor carpi radialis and humeroantebrachialis) flex the elbow joint, and the dorsal ones (e.g., triceps and brachioradialis) extend it (figs. S3 and S4). In this way, *Acanthostega* more closely resembles extant finned sarcopterygians than extant tetrapods. *Acanthostega* is also more similar to the fishes in having relatively little leverage for elbow joint lateral rotation, increasingly so as the elbow is rotated laterally away from neutral position (Fig. 3K).

Thus, muscle leverage around the shoulder joint of *Acanthostega* is more fish-like, with relatively small leverage in lateral rotation but with intermediate leverage in retraction. In combination with the relatively small ROM in humeral long-axis rotation and an elbow in which flexion/extension ROM is dominant, we infer that these data for *Acanthostega* reflect a forelimb poorly suited for weight-bearing but not necessarily unable to generate terrestrial limb-based movements.

### Carboniferous tetrapod *P. finneyae*

*Pederpes* has been interpreted as having potential adaptations for terrestriality (*15*), but our results for most aspects of forelimb musculoskeletal function were not appreciably different from those of *Acanthostega* (Figs. 2 and 3). In *Pederpes*, the summed muscle moment arms for humeral retraction relative to other directions of movement are small compared with crown tetrapods (Fig. 3, E to G). This difference between stem and crown tetrapods is driven largely by changes in mm. triceps and coracobrachialis longus that, according to electromyography and anatomical studies, play important roles in the stance phase of locomotion in extant tetrapods (*32*, *33*, *46*). We did not include m. triceps coracoideus in our early tetrapod models because early tetrapods lack the osteological correlate associated with this muscle (*21*) (see Materials and Methods for an explanation of how osteological correlates of muscle attachment were used to build the models). In most extant quadrupedal tetrapods including lizards and salamanders, triceps coracoideus is an important humeral retractor [e.g., (*32*, *33*)], and in our extant tetrapod models, this muscle head greatly contributes to humeral retraction leverage (Fig. 4, M and N). M. coracobrachialis longus is another important humeral retractor in lizards (*32*) and is thought to play a similar role in salamanders (*46*); however, in early tetrapods, the more posterior position of the glenoid limits the retraction leverage of this muscle (Fig. 4, K and L). Thus, changes to the pectoral girdle in crown tetrapods [appearance of osteological correlates for triceps attachment (*44*) and anterior migration of the glenoid] may signal increasing involvement of the shoulder in body support and motion.

Leverage of humeral depressors is also similar between *Pederpes* and *Acanthostega*, both absolute and relative to other directions (Fig. 3, D and E). Humeral depressors (“shoulder adductors”), along with hip adductors and elbow/knee extensors, help resist the greater external moments generated by non–belly-dragging (“raised”), sprawling postures on land, especially in larger animals (*24*). Therefore, a sprawling animal as large as *Pederpes* [~65-cm snout-vent length; (*15*)] might have had to exert large depression moments at the shoulder joint to raise its chest above the ground. Although the mass of the adductor musculature cannot be estimated from our data, the absence of a trend toward increasing leverage of humeral depressors between *Eusthenopteron*, *Acanthostega*, and *Pederpes* reinforces the idea that enhanced abilities to support body weight appeared somewhat later during tetrapod evolution, possibly among smaller animals within the crown group (*24*).

One aspect of the shoulder musculature of *Pederpes* does resemble that of crown tetrapods: Muscle leverage for all three swing-phase actions are roughly equivalent, in contrast to the fishes and *Acanthostega* where leverage for humeral elevation exceeds lateral rotation and, often, protraction (Fig. 3). This result might indicate that these muscle groups performed a comparable function in *Pederpes* and crown tetrapods, that of repositioning the limb during swing phase. The increased leverage for humeral lateral rotation in *Pederpes* appears to be driven by the dorsal and posterior expansion of the cleithrum, which moves the origin of the dorsal shoulder muscles, particularly m. deltoideus, farther from the glenohumeral joint (Fig. 5, A to F).

Again, similar to *Acanthostega*, elbow joint ROM in *Pederpes* is greatest in flexion-extension, a by-product of a flattened humerus and small radial and ulnar articular facets (Fig. 5, G to L), which limit lateral rotation (see Materials and Methods). However, relative leverage for elbow extension in *Pederpes* is greater than in any other animal we studied (Fig. 3, H to N), indicating a greater ability to resist external flexor moments at the elbow joint (*11*, *24*). This mirrors elbow joint anatomy and function in *Ichthyostega*, which is characterized by large elbow flexion-extension ROM and well-developed elbow extensor musculature (implied by a large olecranon process), features suggested to facilitate forelimb-driven (crutching) locomotion on land (*9*). However, similar to the fishes and *Acanthostega*, *Pederpes* is reconstructed as having had multiple forearm muscles with extensor leverage rather than a single, specialized one (m. triceps) as extant tetrapods do (figs. S3 and S4).

Thus, similar to *Acanthostega*, the shoulder musculature of *Pederpes* appears to have been less specialized than crown tetrapods for generating vertical force against the substrate and supporting the body against gravity due to its relatively small leverage for humeral adduction and retraction. Yet, its large elbow extensor leverage may have increased its ability to generate and counteract larger limb-substrate forces that might be encountered during locomotion using a sprawling limb posture.

## DISCUSSION

The patterns of joint ROM and muscle leverage recovered here can be summarized into two major functional transitions of the pectoral appendage. The first is from a “benthic fish ancestor” locomotor mode resembling extant lungfish to an “early tetrapod” mode that has no close extant analog. The benthic fish ancestor mode has characteristics we associate with locomotion driven primarily by axial undulation, in which the fins play a role in steering (i.e., relatively little leverage for humeral retraction and lateral rotation, shared by *Latimeria*, *Neoceratodus*, and *Eusthenopteron*; Fig. 3). This mode also includes potential specializations for propping the body on a substrate (small humeral protraction-retraction ROM and relatively large leverage for humeral elevation-depression, shared by *Neoceratodus* and *Eusthenopteron*; Figs. 2 and 3). The second transition is from the early tetrapod locomotor mode to a “plesiomorphic crown tetrapod” mode that has characteristics associated with largely limb-based locomotion and weight support (i.e., greatly increased humeral retraction leverage and a specialized role for mm. triceps in elbow extension, shared by *Dicamptodon* and *Iguana*; Fig. 3 and fig. S5). Possible specializations for fast terrestrial locomotion within crown tetrapods include increased relative leverage for humeral depression and ROM for protraction-retraction (in *Iguana*; Figs. 2 and 3).

Distinguishing characteristics of the early tetrapod locomotor mode seem to result from selective pressures related to generating forces against a substrate and stabilizing joints against torsion. In both *Acanthostega* and *Pederpes*, relative shoulder retraction leverage and elbow flexion-extension ROM are greater than in the fishes (Figs. 2 and 3). On the basis of differences between extant fishes and tetrapods, we infer that interaction between the forelimb and substrate became increasingly important for locomotion in early tetrapods, even in taxa such as *Acanthostega* that likely remained largely aquatic (*9*, *10*, *19*, *28*, *41*). The transition to early tetrapod mode also involved decreased ROM in shoulder and elbow long-axis rotation, which likely served to stabilize the limb and body against torsion, which, in salamanders, results from the vertical component of the ground reaction force (*47*). At the same time, increased ROM in humeral protraction-retraction and elbow flexion-extension supports the idea that these anterior-posterior movements of the shoulder and elbow flexion-extension became an important part of forelimb locomotor function in the earliest stages of tetrapod evolution (*16*). These ROM changes could have limited the utility of the forelimb for steering underwater but might have helped position the limb to generate forces against a substrate. The Carboniferous tetrapod *Pederpes* shows some potential specializations for resisting gravity (relatively large elbow extensor leverage) and coordinated swing phase actions (fairly uniform moment arms of humeral elevators, protractors, and lateral rotators).

Although our sample size necessarily is limited, our results indicate that the earliest steps in tetrapod forelimb evolution were related to limb-substrate interaction and its role in locomotion, whereas adaptations for weight support mainly occurred in more crownward taxa. This is consistent with paleontological and developmental evidence that suggests adaptations in the pectoral appendage preceded those in the pelvic appendage [e.g., (*3*, *10*)] and that unique forelimb-driven gaits were used by some early tetrapods (*8*, *9*). However, in the context of locomotion, trends in pectoral appendicular evolution must be considered in conjunction with the pelvic appendage and the body axis. To perform a lateral-sequence walk, all four limbs must minimally be able to reach the ground and generate enough force to anchor the body against slippage or collapse. During terrestrial locomotion in almost all extant quadrupedal tetrapods, hindlimbs provide most of the propulsion, and forelimbs function primarily in braking (*11*). Thus, leverage and ROM for hindlimb retraction are further potential limiting factors in early tetrapod gaits [as well as sufficient knee/ankle mobility; (*9*)]. Undulation of the body axis is also important for increasing stride length in extant sprawling tetrapods (*37*), and thus, the degree of axial mobility could further influence possible gaits in early tetrapods (*8*, *9*, *17*). Future studies of the pelvic appendage and vertebral column would allow us to test and refine hypotheses about locomotor strategies and abilities among early tetrapods and build a more complete picture of the evolution of limb-based terrestrial locomotion.

We conducted the first rigorous analysis of musculoskeletal function in early tetrapods by building on a foundation of data from extant taxa that phylogenetically bracket the tetrapod fin-to-limb and water-to-land transitions (*21*, *22*). Our results support three stages of forelimb functional evolution: first, a “benthic fish” locomotor mode similar to the pectoral fin of extant lungfish, followed by a unique early tetrapod mode distinct from that of extant sarcopterygian fishes and tetrapods, and, last, by a plesiomorphic crown tetrapod mode resembling “modern” tetrapod forelimb function. The results from the two early tetrapod forelimbs (*Acanthostega* and *Pederpes*) are markedly similar, with constrained shoulder mobility and a moderate increase in muscular capacity for humeral retraction, but not depression (“adduction”). Combined with previous data from *Ichthyostega* (*9*, *10*), this similarity suggests that early tetrapods found unique solutions to certain locomotor trade-offs. The early tetrapod locomotor mode could represent specialization for an intermediate form of locomotion such as submerged or partially submerged walking, unique limb/body kinematics, or a transitional evolutionary or life history stage, such as an extended aquatic juvenile phase (*15*, *19*). Although more specimens and analysis of pelvic and axial anatomy are required to fully explore changes in the tetrapod locomotor system, our results add to a growing body of evidence that early tetrapods occupied a distinct niche driven by musculoskeletal “compromise” imposed by their amphibious habits (*13*, *48*).

## MATERIALS AND METHODS

### Specimens and scanning

Our study taxa were three well-preserved fossils that represent three distinct stages in the fin-to-limb and water-to-land transitions: *E. foordi*, a Late Devonian tetrapodomorph closely related to tetrapods (*27*); *A. gunnari*, a Late Devonian tetrapod that retains many aquatic adaptations (*2*, *28*); and *P. finneyae*, an early Carboniferous tetrapod with some proposed adaptations for terrestrial locomotion (*15*). The fossils were micro–computed tomography (CT) scanned at high resolution (table S1) and segmented semiautomatically in Materialise Mimics (Materialise.com). The material from *Acanthostega* came from four separate specimens, which were scaled by measuring the lengths of common elements where possible and by referring to published reconstructions (*28*) when no common elements were present (table S1).

For comparison, four extant taxa were chosen as representative examples of the two closest sister groups of tetrapods (Actinistia and Dipnoi) and the two major clades of extant tetrapods (Amniota and Lissamphibia) (Fig. 1A). Specifically, the lungfish *N. forsteri* (one of the only three extant genera of dipnoans) was chosen because it has been suggested that the muscle anatomy of this genus is most similar to that of the common ancestor of lungfish and tetrapods [e.g., (*49*, *50*)]. *L. chalumnae* is one of only two extant coelacanth species, both within the same genus and morphologically very similar. *D. ensatus* was chosen because it is one of the largest terrestrial salamanders and has relatively well-developed limbs. The lizard *I. iguana* is a large, terrestrial lizard with a generalized body plan. The extant taxa were scanned using various imaging modalities (table S1) and segmented in Amira-Avizo (Thermofisher.com).

### Musculoskeletal models

Digital 3D skeletal models of the pectoral appendage (including girdle, humerus, radius, and ulna) were built, and maximum osteological ROM of the shoulder and elbow joints was estimated in 3D Studio Max (Autodesk.com). Models were oriented in space using coordinate systems mainly following Gatesy (*51*). First, models were translated so that the glenohumeral joint was located at 0,0,0 in the global coordinate system. Then, the models were rotated until the long axis of the sternum, interclavicle, or vertebral column was aligned with the global *X* axis (anteroposterior), with the posterior end in the positive *X* direction (Fig. 1B). The short axis was aligned with the global *Y* axis (mediolateral), and the dorsal aspect faced in the positive *Z* direction (dorsal). If necessary, the entire model was mirrored across the *XZ* plane so that all models represented a right fin/forelimb.

#### Joint coordinate system and axes of movement

Joint axes and centers of rotation (CORs) were specified following prior protocols (*9*, *50*). To make the shoulder joint, an ellipsoid was fitted manually to the humeral head. Both ellipsoid and humerus were translated so that the proximal end of the humeral head just contacted the center of the glenoid cavity, as determined visually. The *X* axis of the ellipsoid (long-axis rotation) was then aligned with the proximodistal (long) axis of the humerus and its *Y* axis (elevation/depression) aligned with the anteroposterior (long) axis of the humeral head. Since the three axes were orthogonal, the *Z* axis (protraction/retraction) did not need to be specified. To make the elbow joint, a cylinder was manually fitted to the articular surface of the distal humeral condyles. Its *X* axis (long-axis rotation) was aligned with the long axis of the radius and ulna, and its *Y* axis (flexion/extension) was aligned with the humeral condyles. The *Z* axis defined the remaining axis of movement (ulnar/radial deviation). The centroids of the shoulder ellipsoid and elbow cylinder were designated as the joints’ CORs. The radius and ulna were translated so that their articular surfaces just contacted the humeral condyles. Joints and segments were hierarchically linked so that moving or rotating proximal elements affected the distal ones as well.

The configuration of the ancestral sarcopterygian pectoral fin is very different from that of the tetrapod forelimb, so we defined our joint axes to make comparisons as intuitive as possible by accommodating for different “neutral” or starting positions. In both the shoulder and elbow, medial (internal) and lateral (external) rotation refer to rotation about the long axis of the bony segment (i.e., pronation and supination of the humerus or ulna/radius; Fig. 1, G and H). Shoulder joint movements were defined relative to the body axis [protraction/retraction in the anteroposterior direction (Fig. 1C) and elevation/depression in the dorsoventral direction (Fig. 1E)], whereas elbow joint movements were defined relative to the distal humeral condyles [radial/ulnar deviation toward the radial and ulnar condyles (Fig. 1D) and flexion/extension about the long axis of the humeral condyles (Fig. 1F)]. We did not model the wrist for several reasons; first, well-preserved carpals are rare in early tetrapods, second, there are no specifically identified homologous bones in fish, and third, there are few, if any, osteological correlates of muscle attachment distal to the radius and ulna (*21*).

#### Correcting for unpreserved soft tissue

A cartilage correction factor [CCF; sensu Holliday *et al.* (*51*)] was applied to the shoulder and elbow joint of each fossil based on measurements from extant taxa and modified by joint morphology of the individual fossil (for the extant taxa, the preserved joint spaces were maintained from the original scans). CCFs at both joints were estimated at roughly 5 to 10% humerus length, based on preserved joint space in our extant taxa (2 to 12% at shoulder; 0 to 13% at elbow) and on measurements from adult *Alligator mississippiensis* [8 and 9% of the length of humerus and ulna, respectively; (*52*)] (table S2). The CCFs were adjusted as follows based on joint morphology and congruence (i.e., disparity between dimensions of humeral head and glenoid cavity): 5% for high congruence and convex humeral head (*Eusthenopteron*), 7.5% for intermediate congruence and flat humeral head (*Acanthostega*), and 10% for low congruence and concave humeral head (*Pederpes*). The humerus and radius/ulna were translated distally (along the local *x* axis; i.e., the proximodistal axis of the limb segment) according to adjusted CCF.

#### Determining osteological ROM and the neutral pose

Maximum osteological ROM was estimated following Pierce *et al.* (*9*). Briefly, joints were rotated about their COR in 5° increments until visual assessment showed either interpenetration of bones or <50% overlap of articular surfaces. Many structures other than bones limit joint mobility (e.g., cartilage, ligaments, and skin), and recent work (*9*, *53*) has shown that the ex vivo mobility of limb joints with all soft tissue is smaller than osteological ROM. However, limb joints would probably need to maintain a minimum overlap of articular surfaces during normal locomotion to transfer forces between the substrate and the body. A limited amount of joint translation of the limb segment, coupled with other movements, was allowed. Translations were limited to 20% of the minor (short) axis of the articular surface of the distal bone (s) (e.g., *31*). This amount was calculated by fitting an ellipse to the articular surface, measuring its minor axis, and then taking 20% of the result. Translations were only allowed in the local *Y* and *Z* directions; e.g., the humerus was not allowed to translate away from the glenoid along its own proximodistal axis. A “neutral” pose was established by finding the middle of the osteological ROM in one axis (as described above), moving the joint to that position, and then repeating the process iteratively across the axes until the joint was in the middle of the osteological ROM in all three axes (*54*). The middle of the osteological ROM, measured, for simplicity, without translations, was defined as the “neutral pose.”

#### Rationale for modeling muscles with soft tissue attachments

In the extinct tetrapod models, the origins for mm. latissimus dorsi and pectoralis, which are largely soft-tissue attachments, were placed on the basis of the anatomy of those muscles in extant lizards and salamanders and on osteological correlates where present (*21*). To account for their large areas of origin, we modeled both muscles in all tetrapods with two lines of action (anterior and posterior) that converged on a single insertion.

In extant salamanders, the origin of m. pectoralis extends anteriorly to the anterior margin of the (cartilaginous) sternum (*46*) and posteriorly about 33% the length of the trunk (pers. obs.), with the most posterior fibers originating from m. rectus abdominis (*46*). In extant lizards, this muscle usually originates from the sternum and median process of the interclavicle, with the most anterior fibers attaching to the lateral processes of the interclavicle and the most posterior fibers attaching to the posteriormost portion of the sternum (*55*, *56*). Because early tetrapods do not have a sternum, we placed the anterior origin point of m. pectoralis on the posterior lateral wings of the interclavicle (behind the clavicle), based on osteological correlates [(*21*) and references therein; fig. S2, B and C]. The interclavicle was not modeled in *Acanthostega*, but its location was approximated on the basis of published reconstructions (*28*). Similar to its anatomy in extant salamanders, we placed the posterior origin point approximately 33% of the distance from the anterior margin of the pectoral girdle to the posterior margin of the pelvis, or 2.7 cm posterior to the glenoid in *Acanthostega* and 3.8 cm in *Pederpes* (fig. S2, B and C). Had we placed the posterior origin point on the posterior margin of the bony pectoral girdle (as in extant lizards), it would have been more anterior: about 1 cm posterior to the glenoid in *Acanthostega* and 0.5 cm in *Pederpes*.

In *Salamandra*, a medium-sized, terrestrial salamander with well-developed limbs, m. latissimus dorsi extends anteriorly to partially overlap the origin of m. deltoideus from the suprascapular cartilage and posteriorly across three to four vertebrae (of 13 to 15 trunk vertebrae) (*46*). In extant lizards, the origin most commonly extends from the neural spine of the last cervical vertebra anteriorly to the seventh dorsal vertebra and last sternal rib posteriorly [(*56*) and references therein]. No osteological correlates for the origin of m. latissimus dorsi have been reported in extant lizards and salamanders, but scars on the posterior or lateral edge of the cleithrum (a dermal bone not present in extant lizards or salamanders) have been interpreted as the origin of m. latissimus dorsi in *Eusthenopteron* (*27*) and of mm. latissimus dorsi and/or deltoideus in some early tetrapods (*21*). In our extinct tetrapod models, we placed the anterior origin point at the posterior margin of the cleithrum (in the region of the fourth dorsal vertebra), based on osteological correlates (fig. S2, B and C). Had we based its placement on the anatomy of m. latissimus dorsi in lizards, the anterior point could be placed much farther forward since the last vertebra identified as “cervical” lies just anterior to the supracleithrum in *Acanthostega* (*28*). However, in both *Acanthostega* and *Pederpes*, the location of the cervical-dorsal junction is uncertain (*15*, *28*), so we chose to prioritize osteological correlates. Lacking any such correlates for its attachment, the posterior point was placed three to four vertebrae behind the cleithrum (in the region of the seventh dorsal vertebra), similar to its anatomy in extant salamanders and lizards (2.75 cm posterior to the glenoid in *Acanthostega* and 7.0 cm in *Pederpes*; fig. S2, B and C).

#### Estimating muscle leverage

Models were imported into Software for Interactive Musculoskeletal Modeling [SIMM; MotionAnalysis.com (*57*)] in their neutral poses (figs. S1 and S2) to estimate muscle leverage across the osteological ROM. Bones, joint CORs, joint axes, osteological ROMs, and neutral poses were taken from the 3D Studio Max models. The origin and insertion of each muscle crossing the shoulder and elbow joints were placed on the basis of muscle maps (Fig. 5 and fig. S6) informed by our prior work on tetrapod forelimb muscle evolution (*21*). Briefly, this work used hypotheses of muscle homology between extant sarcopterygian fishes and tetrapods (*50*) to analyze osteological correlates of muscle attachment in extinct and extant sarcopterygians using parsimony-based character optimization (*21*). Following Witmer (*58*), a muscle that is present in the extant sister group but not in the outgroup (e.g., triceps coracoideus) was reconstructed only if their associated osteological correlate was present. Cylinders representing body profiles were added to assist in placing muscle attachments in extinct taxa (fig. S2). Via points and wrapping surfaces were constructed to constrain muscles to biologically realistic paths. The leverage of each muscle was recorded across the osteological ROM, except that a maximum of 180° in any axis was imposed to simplify muscle wrapping. We assumed that differences in leverage correspond to differences in torque capacity because we lack data on relative muscle sizes and, thus, force-generating capacities that would influence total joint torque-generating capacities. Until methods are developed that can estimate muscle sizes, this is a defensible assumption.

#### Muscle moment arm analysis

Values for muscle leverage (quantified as moment arms), produced using the PlotMaker function in SIMM, were imported into MATLAB (Mathworks, Natick, MA, USA). Moment arms were normalized by dividing by humerus length (see data file S1 for non-normalized values) to make it easier to compare patterns of moment arm magnitude across species with different sized limbs. Normalizing moment arms by femur length is fairly common [e.g., (*26*, *59*)], but we felt that humerus length was a more appropriate metric for a forelimb study. Of course, any metric used to compare animals with different body plans has limitations. For example, the humerus in the fishes is relatively short compared with appendage length and overall body size, resulting in larger normalized moment arms. Therefore, we focus on patterns of relative moment arms among taxa rather than absolute or even normalized magnitude. Normalized moment arms in each direction were added together to produce plots of summed moment arms in each axis of movement (Fig. 3).

### Sensitivity analysis

An obvious potential source of error for fossil osteological ROM is taphonomic distortion. For instance, the humeri of *Acanthostega* have been suggested to be somewhat dorsoventrally compressed (*60*). We used a 3D modeling software (3D Studio Max, Autodesk.com) to estimate the effect of this compression by fitting an ellipsoid to the humeral head of *Acanthostega*, measuring osteological ROM of the ellipsoid within the glenoid (by the same methods already described), then scaling the ellipsoid by 150% in the dorsoventral direction, and repeating the measurements. The results showed that increasing the dorsoventral dimensions of the humeral head by 50% slightly decreased osteological ROM in elevation/depression (−5°) and protraction/retraction (−15°), while medial/lateral rotation was unaffected (fig. S5). These differences are not great enough to affect our overall conclusions about ROM or moment arms.

Another source of uncertainty is the location of muscle attachments. In general, muscle points were placed in the approximate center of their areas of attachment. Muscles with large attachment areas, such as mm. pectoralis and m. latissimus dorsi, were reconstructed using two lines of action representing anterior and posterior portions of the muscles. We performed a sensitivity analysis in *Acanthostega* and *Pederpes* to assess the effect of variation in the location of muscle points using the origins of the posterior parts of mm. latissimus dorsi and pectoralis, which attach to soft tissue and therefore have an uncertain area of origin (see rationale above). In retraction, where both muscles have the largest moment arms, moving the origin by 1 cm anteriorly and posteriorly changed the mean moment arm by 0.17 cm on average. The greatest change was 0.2 to 0.3 cm (30 to 40%) in the moment arm of m. pectoralis (posterior part) in *Acanthostega.* However, even this difference was not enough to change the patterns of summed moment arms (fig. S7), so we judged that the uncertainty in placing soft tissue attachments is not great enough to affect our conclusions. More precise identification of muscle attachment areas, e.g., using microscopic scarring patterns on bones [e.g., (*19*)], would be required to assess smaller-scale differences between taxa.

## SUPPLEMENTARY MATERIALS

Supplementary material for this article is available at http://advances.sciencemag.org/cgi/content/full/7/4/eabd7457/DC1

## Appendix

View/request a protocol for this paper from *Bio-protocol*.
